# Hidden in Adulthood: What is the Prevalence of Attention-Deficit / Hyperactivity Disorder (ADHD) among adults in Greece?

**DOI:** 10.1192/j.eurpsy.2026.11616

**Published:** 2026-07-31

**Authors:** A. Politi, M. Bakola, A. K. Sakaretsanou, R. Kouznetsov, A. Konstantatou, K. S. Kitsou, G. Charalambous, P. Gourzis, E. Jelastopulu

**Affiliations:** 1 Postgraduate Program in Health Services and Units Management, Frederick University of Cyprus, Nicosia, Cyprus; 2 Department of Public Health, Epidemiology and Quality of Life; 3 University of Patras, Medical School, Patras, Greece; 4 Department of Psychiatry, University of Patras, Medical School, Patras, Greece

## Abstract

**Introduction:**

Attention-Deficit/Hyperactivity Disorder (ADHD) is a common neurodevelopmental condition that persists from childhood into adulthood, often impairing daily functioning. Affects approximately 8.4% of children and 2.5% of adults. Although childhood diagnoses are common, prevalence in adults remains less studied, undetected earlier in life.

**Objectives:**

We investigated the presence of ADHD symptoms and traits among Greek adults who were not diagnosed during childhood or adolescence.

**Methods:**

We conducted a cross-sectional study from January to April 2025 among adults aged 18 years and older. Participants completed an online questionnaire that included socio-demographic characteristics and the ASRS-18 (Adult Self-Report ADHD Scale-18 items), six screening and 12 additional items. Each question asked how often a symptom occurred over the past 6 months, on a 0-4 scale, from never=0 to very often=4. Higher scores indicate greater ADHD severity, enabling assessment of inattentive and hyperactive/impulsive symptoms.

**Results:**

A total of 254 adults, 51.6% women, mean age 35 years participated in the study. Most of the participants held a university degree (52.4%), 44.1% were employed, 17.3% reported a psychiatric diagnosis and 12.6% indicated substance use problems. The mean total score was 21 (SD=13), reflecting low to moderate symptom severity. Age was positively correlated with symptom severity, indicating that older adults reported more intense ADHD symptoms (p < 0.001). Having a psychiatric disease (p < 0.001) and a history of substance use (p = 0.02) were both significantly associated with more severe ADHD symptoms. No significant differences were found between men and women (p=0.75), nor across educational levels (p=0.30).

**Conclusions:**

Our study provides novel insights on the prevalence and associated factors of adult ADHD in Greece. Age, psychiatric history, and substance use were significant predictors of increased symptom severity. These findings highlight the importance of early recognition and the value of incorporating screening tools into clinical and research protocols.

**Disclosure of Interest:**

None Declared

